# Small cell carcinoma of the ovary, hypercalcemic type: a mini review

**DOI:** 10.3389/fonc.2025.1645361

**Published:** 2025-08-15

**Authors:** Marta Tripepi, Ana G. da Costa, Dennis S. Chi, Jorge Lima, João Casanova

**Affiliations:** ^1^ Department of Women and Children’s Health, Clinic of Gynecology and Obstetrics, University of Padua, Padua, Italy; ^2^ Gynecologic Oncology Unit, Obstetrics and Gynecology Service, Department of Surgery, Hospital da Luz Lisboa, Lisbon, Portugal; ^3^ Gynecology Service, Department of Surgery, Memorial Sloan Kettering Cancer Center, New York, NY, United States; ^4^ Department of OB/GYN, Weill Cornell Medical College, New York, NY, United States; ^5^ Obstetrics and Gynecology Service, Department of Surgery, Hospital da Luz Lisboa, Lisbon, Portugal; ^6^ Comprehensive Health Research Centre (CHRC), NOVA Medical School, Faculdade de Ciências Médicas, NOVA Medical School (NMS), Faculdade de Ciencias Medicas (FCM), Universidade Nova De Lisboa, Lisbon, Portugal

**Keywords:** small cell ovarian cancer, hypercalcemic type, hypercalcemia, SMARCA4, ovarian cancer, pregnancy, chemotherapy, surgery

## Abstract

Small cell carcinoma of the ovary, hypercalcemic type (SCCOHT) is a rare and highly aggressive ovarian neoplasm, predominantly affecting young women, often in their second or third decade of life. Despite its distinctive clinical and pathological features, diagnosis is frequently delayed due to overlapping characteristics with other small round blue cell tumors. A hallmark of SCCOHT is the biallelic inactivation of the SMARCA4 gene, which leads to loss of BRG1 protein expression and disrupts epigenetic regulation via the SWI/SNF chromatin-remodeling complex. Unlike many other malignancies, SCCOHT exhibits low mutational burden and diploid karyotype, suggesting that epigenetic dysregulation, rather than genomic instability, is the underlying oncogenic mechanism. Clinically, SCCOHT often presents with nonspecific abdominal or pelvic symptoms and is uniquely associated with paraneoplastic hypercalcemia in up to two-thirds of cases. Diagnosis requires a combination of imaging, laboratory evaluation, histopathology, and immunohistochemistry. Treatment is not standardized but typically involves a multimodal approach, including radical surgery and platinum-based chemotherapy, often with multi-agent regimens. The role of radiotherapy is less well defined but may be considered for local control or palliation. Prognosis remains poor, with high recurrence rates and limited response to salvage therapy. Emerging molecular insights have prompted investigations into targeted therapies and immunotherapy, though clinical data are limited. Given the frequent presence of germline SMARCA4 mutations, genetic counseling is strongly recommended, and ongoing research is essential to improve diagnostic accuracy, personalize treatment, and enhance outcomes for this devastating malignancy.

## 1 Introduction

SCCOHT is a rare and highly aggressive malignancy that affects young women, with peak incidence occurring in their early 20s (around 23–24 years of age). However, cases have been reported in patients as young as 7 months and up to 56 years old (1, 2). According to the 2014 World Health Organization (WHO) classification of tumors of the female reproductive tract, SCCOHT is categorized as a miscellaneous ovarian neoplasm (3). Due to overlapping features with other malignancies, diagnosis can be challenging. Loss-of-function mutations in the *SMARCA4* gene, a key component of the SWI/SNF chromatin remodeling complex, have been implicated in the tumorigenesis of SCCOHT and may represent a potential therapeutic target.

### 1.1 Epidemiology, etiology, and molecular pathogenesis

SCCOHT accounts for less than 0.1% of all ovarian cancers (2). In the literature, the reported incidence is roughly 0.12 cases per 1 million people per year (4). The majority of patients are younger than 40 years, with a median age of diagnosis around 24 years (2). Rare cases have been reported in prepubertal girls (5). In 2014, Witkowski et al. identified germline *SMARCA4* mutations in individuals from multiple families affected by SCCOHT, suggesting a potential hereditary predisposition (6, 7).

Immunohistochemical profiling is essential to differentiate small cell carcinoma of the ovary hypercalcemic type from other small “round blue cell” ovarian tumors. SCCOHT typically exhibits a distinct immunoprofile characterized by the expression of Wilms tumor suppressor gene 1 (WT1), epithelial membrane antigen (EMA), vimentin, and cytokeratins, with negative staining for markers such as inhibin, chromogranin, TTF1, S100A1, and alpha-fetoprotein (AFP) (8).

At the molecular level, recurrent loss of heterozygosity (LOH) at chromosome 19p13.2 (the locus of *SMARCA4)* has been identified in SCCOHT (6). The SMARCA4 gene encodes BRG1, the catalytic subunit of the SWI/SNF chromatin-remodeling complex responsible for the regulation of gene transcription. In SCCOHT, biallelic inactivation of *SMARCA4* results in complete loss of BRG1 expression, which disrupts gene regulation and contributes to oncogenesis (9, 10).

In addition to the loss of SMARCA4, emerging preclinical evidence suggests a potential role in the pathogenesis of SCCOHT for other components of the SWI/SNF complex, such as SMARCB1. Although SMARCB1 mutations are rare in this tumor type, its role as a core structural subunit of the SWI/SNF chromatin remodeling complex raises the possibility that secondary dysfunctions or cooperative epigenetic alterations may contribute to oncogenic deregulation, as observed in other SWI/SNF-deficient malignancies (11).

Moreover, the functional antagonism between the SWI/SNF complex and the Polycomb Repressive Complex 2 (PRC2), particularly the methyltransferase EZH2, has garnered significant interest. EZH2 is responsible for catalyzing the repressive H3K27me3 histone mark, which is often upregulated in SCCOHT models lacking SMARCA4. Pharmacologic inhibition of EZH2 has demonstrated anti-tumor efficacy in preclinical models, including cellular growth arrest, induction of apoptosis, and neuronal differentiation, as well as delayed tumor progression in murine xenografts (12). Notably, EZH2 inhibition was associated with re-expression of SMARCA2, suggesting a possible compensatory epigenetic mechanism. These findings highlight a potential therapeutic vulnerability in EZH2 dependency, or “oncogenic EZH2 addiction,” in the context of SMARCA4 deficiency (13). Based on this promising preclinical data, an ongoing phase II clinical trial of tazemetostat in patients with advanced epithelioid sarcoma with loss of INI1/SMARCB1 will evaluate the clinical relevance of targeting this epigenetic axis in patients with SCCOTH (14). There is another ongoing clinical trial called Tazemetostat Expanded Access Program for Adults With Solid Tumors [NCT03874455].

Auguste et al. reported that these aggressive tumors carry diploid DNA content, an unusual finding for such a highly lethal malignancy. They also demonstrated that SCCOHT tumors have a very low mutation burden and lack mutations in genes most commonly altered across in other cancer types. Collectively, these observations support the hypothesis that SCCOHT is driven primarily by epigenetic deregulation rather than by genomic instability (15).

Gao et al. utilized whole-exome sequencing (WES) and single-cell RNA sequencing to analyze SCCOHT, revealing notable intratumoral heterogeneity and providing insights into the tumor’s immune microenvironment (16). This finding pointed to potential therapeutic targets, including genes involved in cell cycle regulation (e.g. CHEK2, CCNB1, WEE1), as well as distinct tumor-associated macrophage (TAM) subsets (15).

## 2 Diagnostic work-up

### 2.1 Clinical Features

Patients typically present with nonspecific pelvic or abdominal symptoms, which can lead to diagnostic delay. Abdominal pain is the most common initial symptom, often resulting from rapid tumor growth or intratumoral hemorrhage/necrosis. Other frequent signs include abdominal distension, bloating, or a palpable pelvic mass (17). Symptoms due to the compression of an adjacent organ, such as constipation or urinary frequency, may also occur (18). Given the young age of many patients, menstrual irregularities or even acute ovarian torsion can sometimes be the first clinical clue.

A notable clinical feature is paraneoplastic hypercalcemia, which occurs in approximately two-thirds of patients (2). Symptoms of hypercalcemia can include lethargy, nausea, polyuria, polydipsia, constipation, confusion, and, in some cases, pancreatitis or altered mental status (2). Laboratory evaluation typically reveals an elevated serum calcium level with suppressed parathyroid hormone (PTH) consistent with a parathyroid hormone-related peptide (PTHrP)-mediated hypercalcemia. CA-125 levels may also be elevated, but this marker is nonspecific and not a reliable indicator for small cell carcinoma of the ovary hypercalcemic type (7).

### 2.2 Imaging

The initial imaging evaluation often includes pelvic ultrasound (TVUS), which typically reveals a large, unilateral, solid adnexal mass with heterogeneous features and complex cystic components (18).

Cross-sectional imaging, typically computed tomography (CT) or magnetic resonance imaging (MRI) is crucial for disease staging and surgical planning. These modalities help assess tumor size, extension to adjacent organs, the presence of ascites, lymph node involvement, and distant metastases. (7). On CT or MRI, SCCOHT often appears as a large, heterogeneous, solid ovarian mass, sometimes accompanied by peritoneal carcinomatosis and ascites. On MRI, these tumors frequently demonstrate solid components that are iso- to hypointense on T1-weighted images and heterogeneously hyperintense on T2-weighted images, with areas of necrosis or hemorrhage (19). Diffusion-weighted imaging (DWI) typically shows marked restriction with low apparent diffusion coefficient (ADC) values, reflecting high tumor cellularity (20). These MRI features, although not pathognomonic, may help radiologists in suspecting SCCOHT in the appropriate clinical context. Positron emission tomography (PET scan) can also be employed to evaluate for metastatic spread. Approximately 50% of cases present with metastatic disease beyond the ovary, with common metastatic sites, including the peritoneum, lungs, liver, and lymph nodes (18).

SCCOHT lacks pathognomonic imaging features, so differential diagnosis based on imaging features is broad. Other tumors, such as germ cell tumors, lymphomas, and other poorly differentiated ovarian neoplasms, should be considered (19).

In clinical practice, the primary role of imaging is to inform surgical planning and detect metastatic disease. Importantly, any solid ovarian lesion in a young female should be considered suspicious for malignancy and prompt further diagnostic evaluation.

### 2.3 Laboratory and biomarkers

Blood tests are another critical component of the work-up. If an ovarian carcinoma is suspected, serum tumor markers should be evaluated; these include CA125, carcinoembryonic antigen (CEA), alpha-fetoprotein (AFP), beta-human chorionic gonadotropin (b-HCG), and lactate dehydrogenase (LDH) (21).

As mentioned earlier, hypercalcemia can be observed, and finding elevated calcium levels in a young patient with an ovarian tumor is highly suggestive of SCCOHT. Such a finding should prompt the measurement of PTH and PTHrP levels to confirm a paraneoplastic hypercalcemia (21). Early recognition of this distinctive biochemical profile is critical, because it supports the diagnosis and has direct implications for management. Severe hypercalcemia can cause symptoms such as fatigue, confusion, nausea, and cardiac arrhythmias, which require prompt correction (22).

In addition to tumor markers and calcium-related assays, basic laboratory tests should be conducted. This includes a complete blood count (CBC), renal and liver function tests, and electrolytes panels, to assess the patient’s overall status and to identify any comorbid conditions or complications. These comprehensive evaluations help guide subsequent diagnostic steps and inform surgical planning (23).

### 2.4 Histopathology

Histopathological examination remains the gold standard for a definitive diagnosis of SCCOHT.

In most cases, the diagnosis is established after an exploratory laparotomy or laparoscopy for an ovarian mass. The resected tumor is examined microscopically, and a panel of immunohistochemical stains is typically required to distinguish SCCOHT type from other ovarian “small round blue cell” tumors. A number of immunohistochemical markers are particularly informative, as summarized in the **Table 1** (8, 24).

**Table 1:** Key immunohistochemical markers in small cell carcinoma of the ovary hypercalcemic type.

Marker	Result in SCCOTH	Diagnostic Role	Sensitivity	Sensibility
Cytokeratins (AE1/AE3)	Positive	Confirms epithelial/carcinomatous nature	81.5%	100%
WT1	Often positive	Nonspecific	NA	75%
Inhibin/Calretinin	Usually negative	Helps exclude sex cord-stromal tumors	NA	NA
OCT3/4	Negative	Rules out dysgerminoma (germ cell tumor)	NA	NA
CD45 (LCA)	Negative	Excludes lymphoma	100%	NA
Synaptophysin/chromogranin	Focally positive in some cases	May suggest neuroendocrine differentiation but is not consistent	100%	NA
SMARCA4 (BRG1)	Loss of expression (in ∼ 95% cases)	*Highly* sp*ecific for* small cell carcinoma of the ovary hypercalcemic type; a diagnostic hallmark	100%	NA
SMARCB1 (INI1)	Retained	Helps distinguish from atypical teratoid/rhabdoid tumors (ATRT), which lose INI1	NA	NA

SMARCA4-(BRG1) immunohistochemistry is particularly critical, revealing a loss of nuclear BRG1 staining in tumor cells while adjacent non-neoplastic stromal cells retain staining (serving as internal controls) (8). In addition, genetic sequencing of *SMARCA4* can aid in confirming the diagnosis and inform genetic counselling (25).

### 2.5 Genetic counseling

Genetic counseling is recommended for all patients diagnosed with SCCOHT, regardless of age or family history, because of the high prevalence of germline mutations in this disease (25).

Comprehensive germline genetic testing should be performed, including full sequencing and deletion/duplication analysis of the SMARCA4 gene. In addition, somatic (tumor) testing can help clarify the nature of SMARCA4 variants. If a pathogenic germline mutation is identified, predictive testing should be offered to at-risk asymptomatic first-degree relatives, ideally alongside appropriate genetic counseling (26).

Although there are no established surveillance guidelines for asymptomatic carriers of SMARCA4 mutations, proactive measures are advisable. These include discussions about reproductive options (such as preimplantation genetic diagnosis or oocyte cryopreservation) and vigilant monitoring for early symptoms of disease (27). Preventive (prophylactic) oophorectomy may be considered in select high-risk cases, but this approach must be weighed against the patient’s fertility goals and psychological well-being (28).

Due to the lack of standardized guidelines, following the most recent guidelines for surveillance in BRCA patients can be useful in patients with known SMARCA4 mutations (29). For women with BRCA1/2 gene mutations, surveillance for ovarian cancer often involves regular TVUS and CA 125 every six months. Testing serum calcium and PTHrP (parathyroid hormone-related peptide) can also be considered (30).

Additionally, in families with a known pathogenic SMARCA4 variant, genetic counseling can guide risk assessment for other related malignancies (for example, rhabdoid tumors) that may also be associated with SMARCA4 loss (6).

## 3 Treatment

### 3.1 Surgery

As with other ovarian malignancies, cytoreductive surgery is a key component in the management of SCCOHT (31).

Given the aggressiveness of the nature of SCCOHT and the typically young age of affected patients, surgical decisions must be individualized, considering the patient’s age, desire for fertility preservation, and extent of disease.

In many cases, the tumor is confined to a single ovary at the time of diagnosis (17), which raises the question of whether a conservative surgical approach could be appropriate (32, 33). In selected Stage I cases involving young women who wish to preserve fertility, surgeons have occasionally attempted to remove only the affected ovary while sparing the uterus and contralateral ovary. However, recurrence rates remain high even in early-stage disease, and most experts advocate for comprehensive surgical management (34). Notably, the largest series published to date observed worse survival outcomes in patients who underwent fertility-sparing surgery (16).

For patients who have completed childbearing or who do not desire fertility preservation, standard surgical management is a comprehensive staging operation. This typically involves removal of the affected ovary and fallopian tube, total hysterectomy with bilateral salpingo-oophorectomy, omentectomy, and pelvic and para-aortic lymphadenectomy (25). In general, the risk of lymph node metastasis is lower in non-serous ovarian cancers (such as SCCOHT) than in high-grade serous carcinomas. However, when nodal metastases do occur in these tumors, their location in unpredictable. Additionally, many non-serous ovarian tumors are relatively chemoresistant (35), raising concerns about the effectiveness of chemotherapy against residual nodal disease. For these reasons, Takeshima et al. have advocated for performing systemic lymphadenectomy even for non-serous tumors (36).

For patients presenting with advanced-stage disease, the surgical team must evaluate whether an optimal cytoreduction (removal of all visible tumor) can be achieved. If complete cytoreduction is attainable, primary debulking surgery is usually preferred (25).

If the disease burden is too extensive for upfront complete resection or the patient’s condition is poor, neoadjuvant chemotherapy followed by interval debulking surgery may be considered. Current data suggest that, when complete resection is ultimately achieved, survival outcomes after interval cytoreduction are not significantly worse than those after primary surgery (29). Interestingly, in contrast to epithelial ovarian cancers, a study by Nasioudis et al. found that even achieving no gross residual disease did not significantly improve survival in patients with Stage II–III SCCOHT (37).

Intraoperative frozen section is a valuable tool for confirming malignancy during surgery, especially in cases suspected to be early-stage ovarian cancer. If carcinoma is confirmed on frozen section, the surgeon can proceed with comprehensive staging during the same operation, including peritoneal biopsies and lymphadenectomy as needed (38).

### 3.2 Systemic therapy

Because SCCOHT is highly aggressive, even patients with FIGO Stage I disease are typically treated with adjuvant chemotherapy to address presumed microscopic metastases (5, 16).

There is no single established chemotherapy protocol for SCCOHT, but nearly all reported regimens incorporate a platinum-based agent (cisplatin or carboplatin) as the backbone (**Table 2**) (39). Platinum drugs are effective against many aggressive ovarian cancers and have formed the cornerstone of SCCOHT treatment since the earliest reported case (23). Various combination regimens have been used, often extrapolated from protocols for other high-grade malignancies. One common approach has been to treat SCCOHT similarly to small cell lung carcinoma by using a combination of cisplatin (or carboplatin) and etoposide (5, 26). Notably, in the series by Harrison et al., all patients who achieved long-term survival had received chemotherapy regimens containing cisplatin and etoposide (39). This platinum–etoposide combination is logical given the “small cell” histology of the tumor and has frequently been augmented with a third agent, such as cyclophosphamide.

**Table 2:** Chemotherapy regimens described in the literature on small cell carcinoma of the ovary hypercalcemic type.

Authors	Year	N. patients	Median age	Type	Treatment	Regimen details	ORR
Young et. al	1994	150	23.9y	Original research	PA	PA	NA
Callegaro-Filho et al.	2015	47	30y	Original research	SX 100% ADJ UNKNOWN 8.5 % SX + CHT 74.5% SX + CHT + RT 8.5%, SX+CHRT 2.1%, SX, no ADJ 4.2%.	CE 38.5 % VPCBAE 25.6% CP 17.9% CPB 2.6% BEP 7.7% CPAE 2.6% CE+CP 2.6% HDCT 2.6%	14.9 months
Blanc-Durand et al.	2020	44	33.3y	Original research	CRS 100% CRS + CHT 86.4%, CRS + CHT + RT 47.7%	PAVEB 86.4% If complete response: CARBOPEC or ICE + ASCHT 68.2% Second-line therapy: T 15.9 % T- B 6.8% Anti-PDL1 9.1%	25.7 months
Nasioudis et al.	2018	469	39Y	Original research	No CRS 17.3 % Only CHT 11.1% CRS + CHT + RT 6.2% CRS + RT 0.2 %	PA	13.2 months
Harrison et al.	2005	17	34.5y	Original research	CRS + CHT 100 % CRS + CHT + RT 41.2 %	BEP 47.1 % CP 23.5% CE + CP 17.6% CAP: 11.8%	PA
Pautier et al.	2007	27	25y	Original research	CRS 74.1% SX 25.1% CHT 100%	PAVEB 100% PAVEB + CARBOPEC + ASCHT 37.0%	PA
Senekjian et al.	1989	5	20y	Original research	SX+CHT+ RT 60% SX + CHT 40%	VPCBAE 100%	NA

CRS, cytoreductive surgery; SX, others surgical procedures; NA, not available; PA, partially available; RT, radiotherapy; CHT, chemotherapy; CHRT, chemoradiotherapy; CE, cisplatin/carboplatin and etoposide; VPCBAE, vinblastine, cisplatin, cyclophosphamide, bleomycin, doxorubicin, and etoposide; CP, carboplatin and paclitaxel; CPB, carboplatin, paclitaxel, and bevacizumab; BEP, bleomycin, etoposide, and cisplatin; CPAE, cisplatin, cyclophosphamide, doxorubicin, and etoposide; CE+CP, cisplatin and etoposide followed by paclitaxel and carboplatin; CAP, cisplatin, adriamycin, and cyclophosphamide; HDCT, high-dose chemotherapy (induction with carboplatin and paclitaxel, cyclophosphamide, etoposide, and cisplatin, followed by hyper-fractioned cyclophosphamide, doxorubicin, and vincristine, followed by autologous stem cell transplant); PAVEP, cisplatin, doxorubicin, etoposide, and cyclophosphamide; CARBOPEC, carboplatin, etoposide, and cyclophosphamide; ICE, ifosfamide, carboplatin, and etoposide; AHSCT, autologous stem-cell transplant; T, taxane; T-B, taxane + bevacizumab.

In the absence of disease-specific treatment guidelines, clinicians have sometimes adopted regimens used for more common ovarian cancers (e.g., high-grade serous carcinoma) (23). This practice has led some to use a platinum-plus-paclitaxel chemotherapy regimen, although there is no clear evidence that this approach is superior to platinum plus etoposide in SCCOHT (39).

Additionally, various intensive multi-drug regimens have been explored in SCCOHT. One such regimen is VPCBAE, which consists of vinblastine, cisplatin, cyclophosphamide, bleomycin, doxorubicin, and etoposide. This protocol was evaluated by Senekjian et al. in a small series: out of five patients treated, three remained disease-free after six cycles of VPCBAE, while the other two (who had residual pelvic disease) showed objective responses (40).

Another aggressive regimen that has been investigated is PAVEP (cisplatin, doxorubicin, etoposide, cyclophosphamide, and bleomycin). In a prospective study, Pautier et al. reported that this dose-intensive chemotherapy, administered after radical cytoreductive surgery, was associated with encouraging survival outcomes, particularly when followed by high-dose chemotherapy with autologous stem cell support (41). More recently, other intensive regimens have been explored, including ICE (ifosfamide, carboplatin, etoposide) and various high-dose chemotherapy approaches. In practice, given the lack of a clear standard, many centers employ a “whole agent” chemotherapy strategy, using multiple active drugs either in succession or in combination (31). A systematic review of 306 SCCOHT cases noted that although chemotherapy protocols varied widely, virtually all patients received a platinum drug plus one or more additional agents (7).

High-dose chemotherapy with autologous stem cell transplantation (HDC/ASCT) has also been explored as a consolidation strategy for SCCOHT in first remission (41). This approach is analogous to treatments used in high-risk germ cell tumors and aggressive pediatric solid tumors. While HDC/ASCT might prolong remission in some patients, its benefit remains unproven due to the rarity of the disease and the absence of controlled trials (41).

Recent studies have highlighted the expression of PD-L1 and tumor-infiltrating immune cells (TILS) in SCCOHT, suggesting a potential role for immune checkpoint inhibition (42, 43). Regarding the use of immunotherapy, there are currently two ongoing clinical trials that are both using pembrolizumab (monoclonal antibody anti PD-L1). One clinical trial [NCT 05368207] will primarily evaluate progression-free survival (PFS), determined through CT scan (14). The other clinical trial is a multicentric non-randomized Phase II trial [NCT04602377], which aims to evaluate the complete response rate of the disease following administration of pembrolizumab in combination with etoposide–cisplatin-based chemotherapy.

### 3.3 Radiotherapy

The role of radiation therapy (RT) in SCCOHT is not well defined, but it may be considered in both adjuvant and palliative settings (39, 44). In patients who experience relapse after complete surgery and intensive chemotherapy, pelvic recurrence is common, making pelvic RT a potential option (39).

Callegaro-Filho et al. observed a trend toward improved survival in patients receiving RT, either as adjuvant or salvage therapy (45).

Historically, whole abdominal radiotherapy (WAR) was used post-surgery to target microscopic disease (5, 39). There have been isolated reports where WAR (or whole-pelvic radiotherapy) combined with chemotherapy resulted in prolonged survival, suggesting a possible benefit for local control (46). Given its high toxicity, especially in young patients, WAR is no longer routine (7, 47).

Some case reports have even suggested that integrating radiation with chemotherapy in the initial treatment (concurrent chemoradiation) might improve local control. In summary, radiation therapy is used as a consolidative treatment for localized residual disease or for palliation of symptoms, but its impact on overall survival (OS) in SCCOHT remains unclear and is likely limited (46).

### 3.4 Treatment during pregnancy

The management of ovarian malignancies during pregnancy is a complex clinical scenario, balancing maternal oncologic treatment with fetal safety. Although epithelial ovarian cancers are more common, rare and aggressive neoplasms, such as SCCOHT, pose unique challenges, including hypercalcemia-related risks (48).

Early diagnosis is often prompted by abdominal symptoms or incidental findings during prenatal imaging. If patients wish to continue the pregnancy, surgical staging can be performed at any gestational age; however, the second trimester is generally preferred to minimize fetal risk while optimizing maternal outcomes (42). Given SCCOHT's aggressive nature, prompt cytoreduction surgery followed by adjuvant chemotherapy is generally recommended (49). Data on chemotherapy safety during pregnancy remain limited, and treatment decisions should be individualized. Chemotherapy is contraindicated in the first trimester of gestation, as early exposure has been associated with a 10-20% risk of major malformations (50). Chemotherapy is also not recommended beyond 35 weeks of gestation; a 3-week interval between the last chemotherapy cycle and delivery is important to allow for recovery of maternal and fetal bone marrow (51). Chemotherapy regimens often include platinum-based combinations, such as cisplatin and etoposide or multi-agent protocols, like VPCBAE (51, 52). Etoposide, despite myelotoxicity, appears relatively safe in pregnancy, though evidence is limited (53).

Multidisciplinary care involving gynecologic oncologists, maternal-fetal medicine specialists, and neonatologists is essential to tailor treatment plans and optimize both maternal and fetal outcomes.

## 4 Prognosis

Data from the literature shows that the prognosis of SCCOHT is generally poor. Reported five-year overall survival for patients with FIGO stage I was 51% (95% CI: 35–75), and 24% (95%:14–40) for patients with FIGO stages II, III, and IV combined (2, 7, 39).

Several prognostic factors have been identified, including the stage of disease at diagnosis, the volume of residual tumor after cytoreductive surgery, the SMARCA4 mutation status, and the presence of hypercalcemia. Several studies have shown that early-stage disease (stage IA) has better outcomes when treated aggressively (39).

Vigilant surveillance is recommended after initial treatment in all cases of SCCOHT due to the high risk of relapse (16).

In a comparative analysis, Hu et al. found that for all small cell ovarian cancer (not only SCCOHT), OS is significantly worse than that for high-grade serous ovarian carcinoma (HGSOC). Median OS was 9.0 months in the small cell ovarian cancer group and 58.0 months in the HGSOC group (54). Additionally, one retrospective study noted several independent predictors of poorer survival in SCCOHT, such as older age at diagnosis, advanced stage, incomplete surgical resection, administration of radiotherapy (likely reflecting more advanced disease), absence of chemotherapy, multiple tumor foci, larger tumor size, and bilateral ovarian involvement (43, 55).

### 4.1 Recurrence

Recurrence rates are very high in SCCOHT; for example, Estel et al. reported a recurrence rate of up to 65.4% in published cases (43).

Recurrent SCCOHT is typically aggressive, with short-lived responses to second-line chemotherapy (31). Nonetheless, some patients have achieved temporary disease control with various salvage regimens, so chemotherapy may still be used in a palliative context. Common salvage regimens have included combinations, such as cyclophosphamide/doxorubicin/vincristine, carboplatin/paclitaxel, or topotecan, though their efficacy is quite limited (26).

Emerging targeted therapies are being explored for recurrent SCCOHT. One case report described a durable partial response to the combination of camrelizumab (an anti–PD-1 checkpoint inhibitor) and apatinib (a VEGFR-2 inhibitor) in a patient with recurrent SCCOHT harboring a SMARCA4 mutation (43). Immunotherapy approaches, particularly immune checkpoint inhibitors, are also under investigation, with ongoing clinical trials assessing their efficacy in this rare cancer (26).

Given the lack of robust clinical data, the management of recurrent SCCOHT should be individualized and ideally undertaken within clinical trials at specialized centers.

## 5 Discussion

SCCOHT is an exceptionally aggressive ovarian tumor that predominantly affects young women (1). Its nonspecific clinical presentation and the overlap of its features with those of other ovarian malignancies often results in delayed diagnosis and missed opportunities for early intervention. Significant progress has been made in understanding the molecular pathogenesis of SCCOHT —most notably, the identification of SMARCA4 inactivation as a key driver—but unfortunately, these insights have not yet translated into standardized or highly effective therapies (26).

Management of SCCOHT remains challenging, typically involving aggressive multimodal treatment including radical surgery and intensive chemotherapy. Radiotherapy may have a role in select cases, but its benefits are not clearly defined. Even with the best efforts, recurrence is common, and the overall prognosis is poor, especially for patients with advanced-stage disease (26, 46). The absence of specific biomarkers and the tumor’s rapid progression further complicate long-term management and follow-up.

Given the rarity, most treatment recommendations are based on small case series or are extrapolated from regimens for other malignancies. This reality underscores the urgent need for international collaboration, prospective research, and the development of targeted therapies specifically for SCCOHT. In the meantime, heightened awareness among healthcare providers, timely incorporation of genetic counseling, and individualized patient-centered care are essential for improving outcomes. Although SCCOHT remains a formidable clinical challenge, continued research efforts and a multidisciplinary approach offer hope for better therapies and improved outcomes in the future for affected patients and their families.
